# The *Plasmodium* LAP complex affects crystalloid biogenesis and oocyst cell division

**DOI:** 10.1016/j.ijpara.2018.09.002

**Published:** 2018-12

**Authors:** Sadia Saeed, Annie Z. Tremp, Johannes T. Dessens

**Affiliations:** Pathogen Molecular Biology Department, Faculty of Infectious and Tropical Diseases, London School of Hygiene & Tropical Medicine, Keppel Street, London, UK

**Keywords:** LCCL protein, Crystalloid, Sporogony, Transmission, Malaria, Mosquito

## Abstract

Malaria parasite oocysts located on the mosquito midgut generate sporozoites by a process called sporogony. *Plasmodium berghei* parasites express six LCCL lectin domain adhesive-like proteins (LAPs), which operate as a complex and share a localisation in the crystalloid – an organelle found in the ookinete and young oocyst. Depletion of LAPs prevents crystalloid formation, increases oocyst growth, and blocks sporogony. Here, we describe a LAP4 mutant that has abnormal crystalloid biogenesis and produces oocysts that display reduced growth and premature sporogony. These findings provide evidence for a role of the LAP complex in regulating oocyst cell division via the crystalloid.

## Highlights

• Fusion of GFP to *Plasmodium berghei* LAP4 causes abnormal crystalloid formation.
• LAP4/GFP oocysts have reduced size.
• LAP4/GFP oocyst populations show earlier sporulation dynamics.
• LAP4/GFP sporozoites are not transmitted by mosquito bite.

## Graphical abstract

**Figure:**
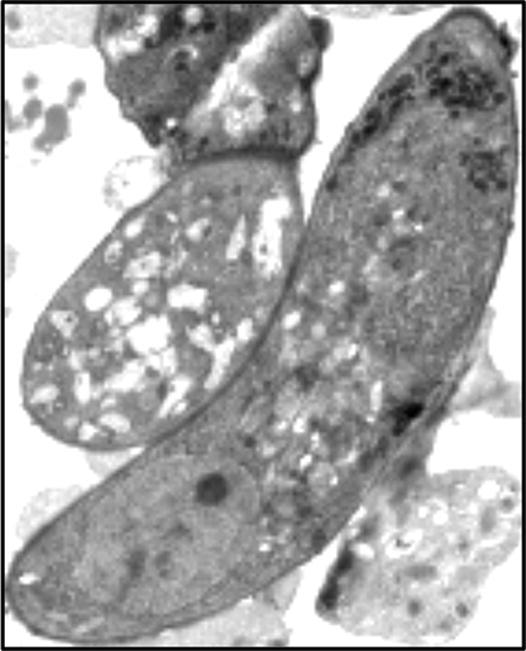

Malaria remains a major human parasitic disease and curbing its transmission by mosquitoes is considered an essential part of successful malaria control and eradication programmes. Parasite transmission is initiated by the uptake of sexual stage precursor cells (gametocytes) from the vertebrate host with the blood meal of a feeding mosquito, setting off a rapid process of gametogenesis and fertilisation. The resulting zygotes undergo meiosis and go on to transform into motile elongated forms called ookinetes, which traverse the mosquito midgut epithelium and then round up to form the oocysts. In the ensuing weeks, these young oocysts grow and divide by a multiple fission-like process known as sporogony to generate thousands of progeny cells named sporozoites. After egress from the oocyst, the motile sporozoites invade and inhabit the salivary glands and are transmissible to new hosts by mosquito bite to initiate new malaria infections (Greenwood et al., 2008).

Development of malaria parasites in the mosquito midgut is accompanied by substantial parasite death (Barillas-Mury and Kumar, 2005). Successful malaria transmission therefore critically depends on the parasite multiplication step during sporogony. Successful sporogony and sporozoite transmission require expression of a family of six conserved modular proteins named LCCL lectin domain adhesive-like proteins (LAPs). The LAPs are almost exclusively found in apicomplexan parasites and possess multiple adhesive-like domains implicated in protein, lipid and carbohydrate binding (Dessens et al., 2004, Pradel et al., 2004), including the ‘LCCL’ domain, a conserved protein module first identified in the founding proteins *Limulus* clotting factor C; cochlear protein Coch-5b2; and lung gestation protein Lgl1 (Trexler et al., 2000). In *Plasmodium* the LAPs form a protein complex (Simon et al., 2009, Tremp et al., 2017). Disruption in *P. berghei* of any of the six *lap* genes, or simultaneous disruption of multiple *lap* genes, gives rise to very similar loss-of-function phenotypes typified by a failure of the oocyst to undergo cytokinesis and produce sporozoites (Claudianos et al., 2002, Raine et al., 2007, Carter et al., 2008, Lavazec et al., 2009, Saeed et al., 2015). Another important shared feature of the LAPs is their subcellular localisation in the crystalloid as was demonstrated by GFP tagging of the six LAPs in transgenic *P. berghei* parasites (Carter et al., 2008, Saeed et al., 2010, Saeed et al., 2013, Dessens et al., 2011). Crystalloids are conserved in the genus *Plasmodium* and they appear in transmission electron microscopy as large clusters of small spherical vesicles (Meis and Ponnudurai, 1987). The organelles form in developing ookinetes by a process of active microtubule-dependent transport and assembly of the subunit vesicles (Saeed et al., 2015). The process of biogenesis completes with the multiple crystalloids present in the ookinete merging during oocyst transition, resulting in a single large crystalloid organelle in each oocyst (Saeed et al., 2015). Disruption of LAP1 or LAP3 expression in *P. berghei* was shown to completely abolish formation of crystalloids (Carter et al., 2008, Saeed et al., 2015, Tremp et al., 2017), pointing to a role of the LAP complex in crystalloid biogenesis. Further support for such a role was obtained using a mutant parasite line in which the LCCL domain was removed from LAP3; although this parasite could form normal crystalloids and displayed normal sporozoite development and transmission, the formation of the organelle in the ookinete was markedly delayed (Saeed et al., 2015).

The role of the LAPs in sporogonic development remains a matter of speculation. Given the ‘extracellular’ features of the LAPs (i.e. the presence of an endoplasmic reticulum (ER) signal peptide and adhesive-type domains), and the presence of domains also commonly found in immunity-related molecules of metazoans, it has been suggested that the LAPs are virulence factors involved in immune evasion in the mosquito (Claudianos et al., 2002, Delrieu et al., 2002, Pradel et al., 2004, Trueman et al., 2004). Based on cytological features of LAP null mutant oocysts, particularly the lack of cytokinesis, others have speculated that the LAPs could have a role in oocyst cell cycle regulation (Raine et al., 2007). For either of these hypotheses, the supporting evidence is at best circumstantial, and little progress has been made on this question for over a decade. In this paper, we describe a LAP modification that not only results in a novel, abnormal crystalloid phenotype, but that also has a remarkable impact on oocyst and sporozoite development that provides compelling supporting evidence for a link between LAP expression, crystalloid biogenesis and oocyst cell division.

This study started with the discovery that our parasite line expressing GFP-tagged LAP4 (PBANKA_1319500) (Saeed et al., 2013) was not transmissible despite developing sporozoites, prompting a re-examination of its development in mosquitoes. Western blot analysis of LAP4/GFP ookinetes with anti-GFP antibodies detected a single prominent band of approximately 200 kDa, corresponding to the LAP4::GFP fusion protein (calculated mass 210 kDa) (Fig. 1A), indicating that the GFP-tagged LAP4 protein is stably expressed in this life stage. In confocal microscopic examination, ookinetes and young oocysts of LAP4/GFP parasites possessed discrete fluorescent areas that co-localised strongly with pigment clusters, which is typical of crystalloid proteins (Fig. 1B). LAP4::GFP fluorescence observed in these life cycle stages was fully consistent with the multiple ‘sub’ crystalloids in the ookinete merging into a single large crystalloid at oocyst transition (Fig. 1B) (Saeed et al., 2015). In addition, the fluorescence distribution in LAP4/GFP ookinetes was clearly distinct from the ER localisation of truncated and dysfunctional LAP1::GFP molecules that give rise to a LAP null mutant phenotype (Carter et al., 2008, Tremp et al., 2017). However, comparison with LAP3::GFP-expressing parasites revealed that the fluorescent areas in the LAP4/GFP parasites were less focal (100%, *n* = 50) (Fig. 1B, C). The same was observed in a second, independent clone of parasite line LAP4/GFP that was generated in the same way (data not shown).

**Fig. 1:**
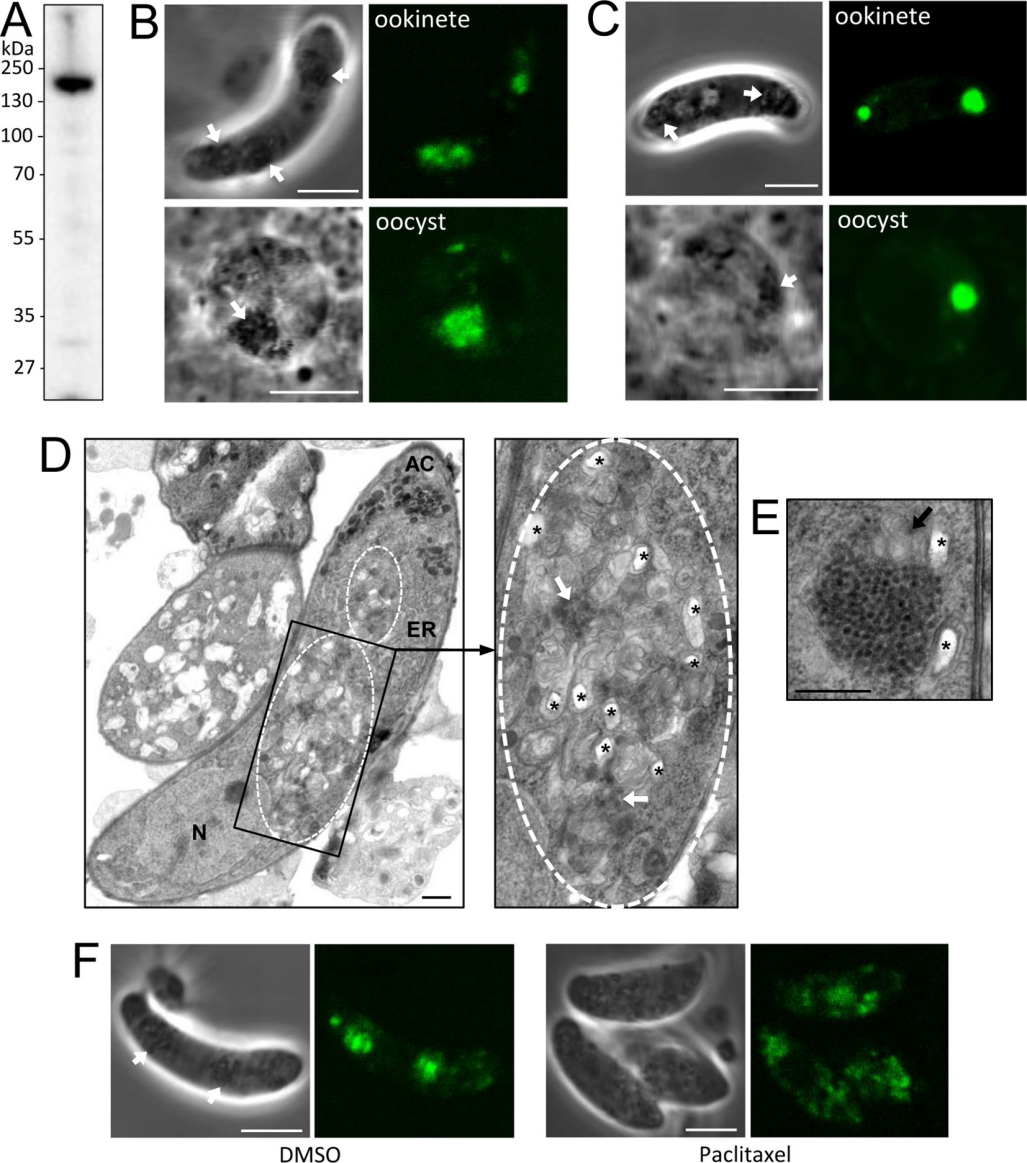
LAP4/GFP *Plasmodium berghei* parasites possess abnormal crystalloids. (A) Western blot of purified ookinetes of parasite line LAP4/GFP using anti-GFP antibodies, revealing a main signal of approximately 200 kDa corresponding to the LAP4::GFP fusion protein. (B) Confocal images of a mature ookinete and young oocyst of parasite line LAP4/GFP and (C) LAP3/GFP. Fluorescence co-localises with pigment clusters (arrows). Scale bar = 5 μm. (D) Transmission electron micrograph of a mature LAP4/GFP ookinete, showing abnormal crystalloid-like structures (delineated by white dashed lines) embedded within the endoplasmic reticulum (ER). Also indicated are the nucleus (N) and apical complex (AC). The black boxed region is enlarged and shows small clusters of crystalloid vesicles (white arrows) amongst abundant membranous material. Asterisks mark vesicles containing hemozoin pigment. Scale bar = 500 nm. (E) A normal crystalloid in a LAP3/GFP ookinete composed a of large cluster of subunit vesicles and small amount of membranous material (black arrow). Scale bar = 500 nm. (F) Confocal images of LAP4/GFP ookinetes formed in the presence or absence (DMSO) of paclitaxel (Saeed et al., 2015). Scale bar = 5 μm. Parasite samples were assessed, and images captured, on a Zeiss LSM510 confocal microscope using a 100× objective. Parasites were prepared for electron microscopy as described (Saeed et al., 2015) and examined on a Jeol 1200EX Mark II transmission electron microscope and digital images recorded with a 1 K 1.3 M pixel High Sensitivity AMT Advantage ER-150 CCD camera system. Animal work was conducted under UK Home Office license and approval in accordance with the United Kingdom Animals (Scientific Procedures) Act 1986 implementing European Directive 2010/63 for the protection of animals used for experimental purposes. All methods were carried out in accordance with relevant guidelines and regulations and approval was obtained from the London School of Hygiene & Tropical Medicine Animal Welfare Ethics Review Board. Experiments were conducted in 6–8 weeks old female CD1 mice, specific pathogen-free and maintained in filter cages. Animal welfare was assessed daily and animals were humanely killed upon reaching experimental or clinical endpoints.

Electron microscopic examination of LAP4/GFP ookinetes revealed that the cells did not possess normal crystalloids, but instead had abnormal crystalloid-like structures that are absent in wild type and LAP null mutant ookinetes (Fig. 1D). Whilst normal crystalloids are made up of large clusters of subunit vesicles that can often be observed associated with small amounts of membranous material (Fig. 1E), the unique structures observed in LAP4/GFP ookinetes contained large amounts of membranous material interspersed with small amounts of crystalloid subunit-like vesicles (Fig. 1D). Apart from the crystalloids, other structures in the ookinetes (e.g. nucleus, endoplasmic reticulum, apical complex) appeared normally formed (Fig. 1D), indicating that the cells were not stressed or otherwise affected in fitness. These combined observations pointed to a defective crystalloid biogenesis process in LAP4/GFP parasites, which was unintentionally caused by the carboxy-terminal fusion of GFP to LAP4. Treatment of LAP3/GFP zygotes with the microtubule drug paclitaxel has been shown to impede crystalloid assembly (Saeed et al., 2015) and the same treatment of LAP4/GFP zygotes interfered with formation of the discrete regions of fluorescence seen in mature ookinetes (Fig. 1F). Thus, the abnormal crystalloid-like structures present in parasite line LAP4/GFP appear to behave like normal crystalloids with respect to their dynamics of biogenesis.

To assess the effects of the crystalloid defect identified in the LAP4/GFP parasite on oocyst and sporozoite development, *Anopheles stephensi* vector mosquitoes were infected and parasite development was monitored in comparison with parasites that form normal crystalloids and display wild type sporogony (parasite line LAP3/GFP expressing LAP3::GFP) (Saeed et al., 2015), and with parasites that fail to form crystalloids and lack sporozoite formation (parasite line LAP3-KO, a LAP3 null mutant) (Saeed et al., 2015). LAP4/GFP parasites produced oocysts in numbers comparable to the other lines (Table 1), indicating that the crystalloid defect had not adversely affected ookinete fitness or infectivity. This is consistent with previous observations that the LAPs are not involved in ookinete infectivity (Raine et al., 2007, Carter et al., 2008, Saeed et al., 2015). However, closer assessment of the crystalloid mutants revealed notable differences in oocyst size. At 14 days p.i., LAP3-KO oocysts had reached a significantly larger size (*P* < 0.0001) than their wild type counterparts (Fig. 2A). This enlarged oocyst phenotype has also been reported for other LAP null mutants (Claudianos et al., 2002, Raine et al., 2007). In contrast, LAP4/GFP oocysts were significantly smaller than control oocysts (*P* < 0.0001) at 14 days p.i., and already lagged significantly in size at 8 days p.i. (*P* < 0.01) (Fig. 2A). Thus, LAP4/GFP parasites display premature oocyst growth arrest.

**Table 1:** Parasite development in *Anopheles stephensi* infected with *Plasmodium berghei* lines LAP4/GFP, LAP3/GFP and LAP3-KO.

Experiment	Parasite line	Mean ± S.E.M. oocyst number 10 dpi^a^ (*n*)^b^	Mean midgut sporozoite number 10 dpi (*n*)	Mean midgut sporozoite number 14 dpi (*n*)	Mean hemocoel sporozoite number 10 dpi (*n*)	Mean hemocoel sporozoite number 14 dpi (*n*)	Mean hemocoel sporozoite number 21 dpi (*n*)	Mean salivary gland sporozoite number 21 dpi (*n*)
1	LAP3/GFP	33 ± 15 (10)	0 (10)	3200 (10)	n/a^c^	n/a	n/a	n/a
	LAP4/GFP clone 1	29 ± 20 (10)	3500 (10)	2200 (10)	n/a	n/a	n/a	n/a
2	LAP3/GFP	45 ± 19 (10)	600 (10)	22,500 (10)	n/a	n/a	n/a	n/a
	LAP4/GFP clone 2	38 ± 21 (10)	8400 (10)	6750 (10)	n/a	n/a	n/a	n/a
3	LAP3/GFP	57 ± 13 (20)	n/a	n/a	0 (20)	1066 (20)	830 (20)	2900 (20)
	LAP4/GFP clone 2	70 ± 16 (20)	n/a	n/a	0 (20)	118 (20)	131 (18)	125 (20)
4	LAP3/GFP	74 ± 21 (20)	n/a	n/a	n/a	n/a	n/a	4530 (35)
	LAP4/GFP clone 1	76 ± 25 (20)	n/a	n/a	n/a	n/a	n/a	145 (40)
	LAP3-KO	81 ± 26 (20)	n/a	n/a	n/a	n/a	n/a	0 (38)

a dpi, days p.i.

b *n*, number of mosquitoes dissected.

c n/a, not assessed.

**Fig. 2:**
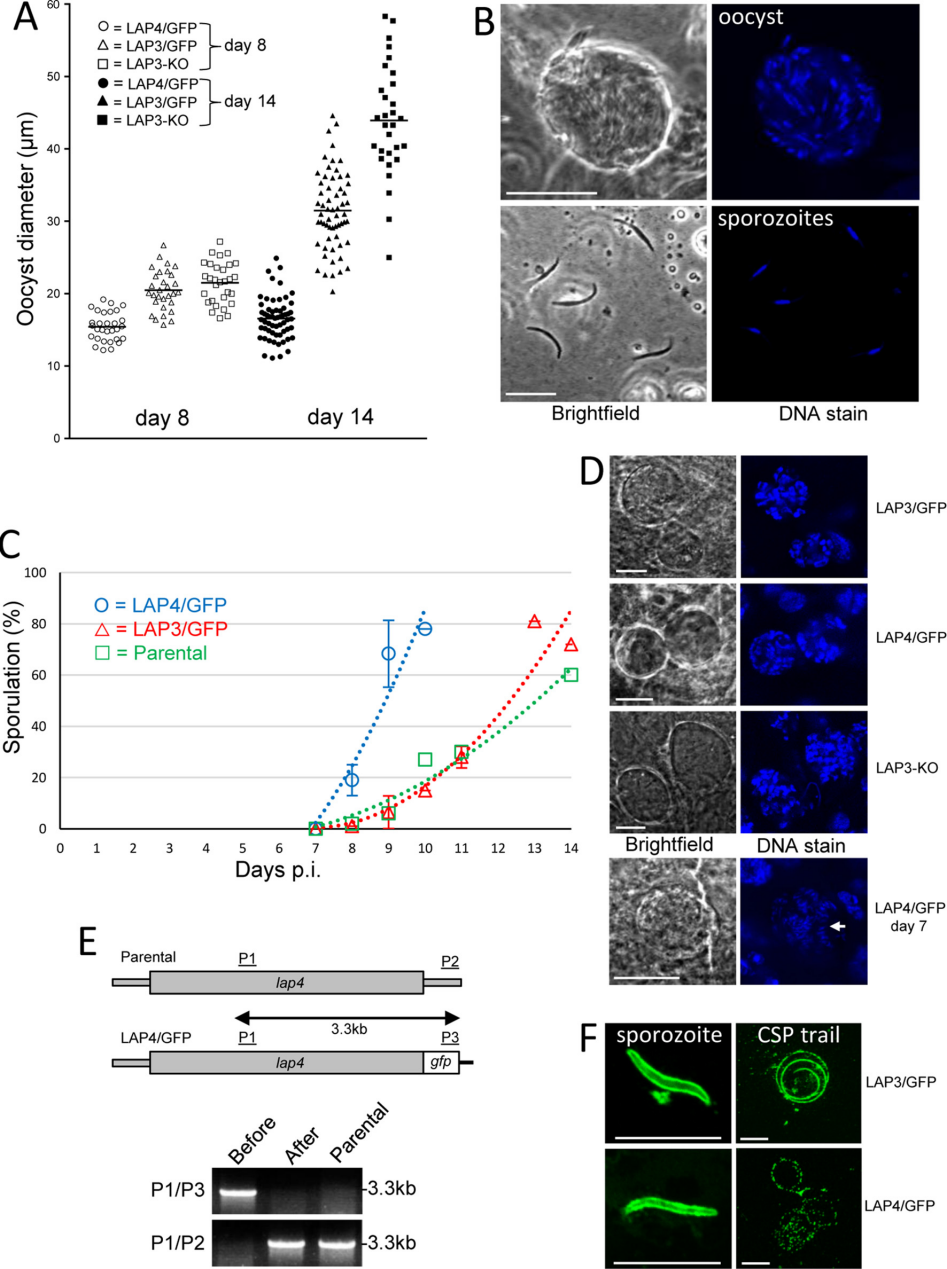
LAP4/GFP *Plasmodium berghei* parasites display abnormal oocyst development and sporozoite infectivity. (A) Oocyst diameter in parasite lines LAP4/GFP (abnormal crystalloids), LAP3/GFP (normal crystalloids) and LAP3-KO (no crystalloids) at 8 days (open symbols) and 14 days (closed symbols) p.i. Horizontal lines denote mean values. Measurements were taken from oocysts from 3 to 5 infected mosquitoes per parasite sample (average 10 oocysts per insect). LAP3/GFP and LAP4/GFP data from day 14 were pooled from two independent experiments using independent clones of LAP4/GFP. (B) Confocal images of a LAP4/GFP sporulating oocyst and released sporozoites. Hoechst DNA stain (blue) labels the nuclei. Scale bar = 10 μm. (C) Time course of sporulation in oocyst populations of parasite lines LAP4/GFP (abnormal crystalloids), LAP3/GFP (normal crystalloids), and parental *P. berghei* (normal crystalloids). Error bars denote the standard errors from replicate data points (LAP4/GFP: three independent experiments and two independent clones used; LAP3/GFP: two independent experiments). (D) Representative confocal images of oocysts from parasite lines LAP3/GFP (normal crystalloids), LAP4/GFP (abnormal crystalloids) and LAP3-KO (no crystalloids). A day 7 oocyst of LAP4/GFP is shown containing condensed elongated nuclei (arrow), indicative of sporozoite budding. DNA is labelled with Hoechst stain (blue). Scale bar = 10 μm. (E) Diagnostic PCR for the presence of modified (primers P1/P3) and unmodified (primers P1/P2) *lap4* alleles in parasite line LAP4/GFP before and after sporozoite transmission, and in parental *P. berghei*, as described (Saeed et al., 2013). A schematic diagram of the modified and unmodified *lap4* alleles is shown with primer sites indicated. (F) Representative images of sporozoites and circumsporozoite trails produced by sporozoites from LAP3/GFP and LAP4/GFP parasites, visualised by immunofluorescence with anti-circumsporozoite primary antibody 3D11 and Alexa Fluor 488 goat anti-mouse secondary antibody. Scale bar = 10 μm. (For interpretation of the references to colour in this figure legend, the reader is referred to the web version of this article.)

In contrast to LAP3 null mutant oocysts that fail to produce sporozoites (Saeed et al., 2015), the large majority of LAP4/GFP oocysts underwent cytokinesis and produced sporozoites of normal morphology and size (Fig. 2B). To investigate the sporulation dynamics, a time course was carried out during which oocysts were examined by light microscopy for visible signs of sporozoite formation. For the first week after parasite infection, oocyst growth and appearance were indistinguishable in all three parasite lines examined and no sporulation was detected. Clear signs of sporozoite formation inside the oocyst were first detected at 8 days p.i., and the proportion of these in the oocyst population increased over time, reaching peak levels (∼80%) around 2 weeks post-infection in both wild type parental and LAP3/GFP parasite lines (Fig. 2C). After reaching these levels, sporulation levels became too difficult to reliably assess, due to sporozoite egress and degeneration of older oocysts. Sporulation in the LAP4/GFP parasites was equally first detected at 8 days p.i., however the oocyst population reached peak sporulation levels several days earlier than in its wild type and LAP3/GFP counterparts (Fig. 2C). Counting sporozoites that were artificially released from oocyst-infected midguts showed that LAP4/GFP oocysts had formed higher numbers of sporozoites than equivalent LAP3/GFP oocyts at 10 days p.i. (Table 1). By 14 days p.i. midgut sporozoite numbers had increased in LAP3/GFP oocyst-infected midguts, but not in equivalent LAP4/GFP oocyst-infected mosquitoes (Table 1). Similar observations were made with a second, independent clone of parasite line LAP4/GFP (Table 1). These collective observations are consistent with the LAP4/GFP oocyst populations sporulating sooner than their wild type and LAP3/GFP counterparts. Assessment of oocyst development using DNA staining revealed no obvious differences in DNA distribution between the three parasite lines examined (Fig. 2D), indicating that the processes of mitosis are not fundamentally different. Nonetheless, at 7 days p.i. we sporadically observed oocysts with condensed and elongated nuclei, indicative of sporozoite budding in the LAP4/GFP parasite (Fig. 2D), consistent with the earlier sporulation dynamics of this parasite line.

Despite the ability of LAP4/GFP oocysts to produce sporozoites (Table 1), salivary gland sporozoite numbers at 21 days p.i. were highly reduced (>10-fold) compared with LAP3/GFP control parasites (Table 1). Moreover, we were repeatedly unable to transmit LAP4/GFP sporozoites to naive mice by mosquito bite (five unsuccessful transmissions in five attempts), while control LAP3/GFP parasites were readily transmitted (two successful transmissions in two attempts). Thus, LAP4/GFP sporozoites are less infective than their wild type counterparts. On one occasion a blood stage infection was obtained in response to LAP4/GFP parasite-infected mosquito bites, but the transmitted parasites possessed an unmodified *lap4* allele (Fig. 2E). These transmitted parasites no longer showed green fluorescence in ookinetes and displayed normal sporulation dynamics (data not shown), indicating that reversion to wildtype had occurred. This was confirmed by sequencing the *lap4* allelle in the revertant, which revealed that it expressed LAP4 no longer fused to GFP. Parasite line LAP4/GFP was generated via single crossover homologous recombination (Saeed et al., 2013), allowing reversion to occur at low frequency. Reversion to wild type confirms that its mutant phenotype was indeed caused by the intended genetic modification of the *lap4* allele. Moreover, the selection for transmission of reverted sporozoites over the non-reverted population supports the notion that LAP4/GFP sporozoites have reduced infectivity compared with wild type sporozoites.

To further investigate the reasons behind the reduced infectivity of LAP4/GFP sporozoites, mosquito hemolymph was collected at 10, 14 and 21 days p.i. This revealed that LAP3/GFP parasite-infected mosquitoes contained markedly higher numbers of hemocoel sporozoites than insects similarly infected with LAP4/GFP (Table 1). In addition, LAP4/GFP sporozoites did not accumulate in the hemocoel (Table 1) as has been observed for other mutant lines incapable of salivary gland colonisation (Kariu et al., 2002, Matuschewski et al., 2002). These observations point to reduced sporozoite egress from the oocyst, or diminished sporozoite survival, in the LAP4/GFP parasite. We also assessed sporozoite motility by visualising trails of circumsporozoite protein (CSP) left behind by gliding sporozoites. Both LAP3/GFP and LAP4/GFP sporozoites had comparable levels of CSP on their surface and produced circular trails (Fig. 2F). However, while the majority of LAP3/GFP sporozoites left continuous trails (77%, *n* = 52), those from LAP4/GFP sporozoites were less pronounced and interrupted (*n* = 30) (Fig. 2F). This is likely to reflect a slower or otherwise anomalous (e.g. start-stop) mode of motility of the LAP4/GFP sporozoites, which could contribute to the observed reductions in sporozoite numbers in hemocoel and salivary glands. These LAP4/GFP phenotypes have similarities with those of parasites depleted of the sporozoite invasion-associated protein 1 (SIAP1), which also displayed reductions in sporozoite egress, salivary gland invasion and continuous gliding, but did not show abnormalities during sporogony (Engelmann et al., 2009). We postulate that the abnormal crystalloid formation in the LAP4/GFP parasite not only disrupts the sporulation dynamics, but also affects the expression of sporozoite proteins, resulting in reduced sporozoite fitness and transmission.

The abnormal crystalloid formation caused by GFP tagging of LAP4 (Fig. 1) was unexpected, because carboxy-terminal fusion of GFP to other LAPs does not result in abnormal sporogony phenotypes (Carter et al., 2008, Saeed et al., 2010, Saeed et al., 2013). One explanation is that in the case of LAP4 the GFP fusion interferes with correct folding. Another explanation is that the GFP fusion has interfered with LAP4 interacting correctly with partner molecules in the LAP complex through steric hindrance. We know from recent work that LAP knockout, or the deletion of specific domains of LAP proteins, impacts on LAP complex formation (Tremp et al., 2017). These same studies also showed that LAP4 co-purifies with LAP5 in GFP pulldown samples of LAP5::GFP-expressing ookinetes without prior in vivo crosslinking (Tremp et al., 2017). The same high affinity LAP4-LAP5 interaction was demonstrated in the reciprocal pulldown of LAP4::GFP-expressing ookinetes (Tremp et al., 2017), indicating that LAP4, despite its fusion to GFP, interacts normally with LAP5. It is also probable that all six LAPs are present in the LAP complex of LAP4/GFP parasites, because the absence of any LAP family members would have resulted in a LAP knockout phenotype, which clearly is not the case. Thus, the effect on the LAP complex caused by the GFP tagging of LAP4 could be subtle. This is not unprecedented; the LCCL domain deletion of LAP3, for example, does not obviously affect LAP complex formation (Tremp et al., 2017), but does nonetheless have a measurable effect on crystalloid biogenesis by slowing down this process (Saeed et al., 2015).

The formation of abnormal crystalloids in LAP4/GFP parasites is accompanied by precocious oocyst growth arrest and cytokinesis within the oocyst population (Fig. 2). This is not a stand-alone observation, but must be viewed in the context of LAP deletions that abolish crystalloid formation and have an almost opposite effect on sporogony by displaying a lack of cytokinesis and delayed oocyst growth arrest (Fig. 2) (Claudianos et al., 2002, Raine et al., 2007, Carter et al., 2008). This makes it unlikely that the effects on oocyst cell division in the LAP4/GFP parasite are caused by non-specific mechanisms, and points instead to a specific role of the crystalloid in sporogony. The LAPs are not expressed at the time of cytokinesis in the oocyst (Carter et al., 2008, Lavazec et al., 2009), suggesting that their role in this process is indirect. In addition, only LAP mutants that fail to form normal crystalloids (i.e. LAP4/GFP and LAP null mutants) display abnormal oocyst cell division, indicating that the crystalloid and sporogony phenotypes are causally linked rather than pleiotropic effects of the LAP modifications. A functional link between crystalloids and sporogony is also supported by null mutants of the crystalloid-resident palmitoyl-acyl-transferase DHHC10, which fail to form crystalloids and sporozoites (Santos et al., 2016). Collectively, these data point to an indirect involvement of the LAPs in sporogony through the LAP-dependent process of crystalloid biogenesis.

In binary fission, and mitotic division generally, cytokinesis is temporally regulated as part of the cell cycle to allow sufficient time for cytoplasmic expansion, DNA replication and nuclear division, ensuring that daughter cells end up the correct size and genetic composition (Mishima et al., 2004, Petronczki et al., 2007, Slavov and Botstein, 2011, Campos et al., 2014, Taheri-Araghi et al., 2015). Less is known about these processes during multiple fission such as sporogony, where cytokinesis occurs only after multiple rounds of growth and mitosis. CSP null mutant oocysts, despite also lacking in cytokinesis, do not grow to an abnormal size (Menard et al., 1997), and the same has been reported for cytokinesis-deficient null mutant oocysts of Ap2-SP (Yuda et al., 2010), demonstrating that increased oocyst growth in the absence of cytokinesis is not a default outcome. This, in turn, indicates that the LAP mutant phenotypes regarding oocyst growth and cytokinesis are causally linked and LAP-specific. In the context of multiple fission, the absence of cytokinesis could allow more rounds of growth/mitosis during sporogony, resulting in larger oocysts that do not sporulate, as observed in LAP null mutants. By analogy, an earlier onset of cytokinesis during sporogony would reduce the number of rounds of growth/mitosis, resulting in smaller oocysts as well as earlier sporulation, exactly as is observed in our LAP4/GFP parasite line (Fig. 2). The combined phenotypes of these distinct LAP mutants thus provide compelling supporting evidence for a role of the LAPs, or a LAP-dependent process, in cell division within the oocyst, particularly with respect to cytokinesis.

Reduced oocyst size has also been observed in parasites depleted of the plant-type cyclin CYC3 (Roques et al., 2015). However, this phenotype was accompanied by marked reductions (>60%) in the proportion of oocysts that reached maturity and sporulated, and sporozoites were normally transmissible (Roques et al., 2015), which is clearly distinct from the LAP4/GFP phenotype reported here. In the human malaria parasite *P. falciparum,* null mutant oocysts of *Pf*CCp3 (orthologue of *Pb*LAP1) and *Pf*CCp2 (orthologue of *Pb*LAP4) undergo seemingly normal sporogony, but generate sporozoites that are unable to reach the salivary glands (Pradel et al., 2004). Whilst these phenotypes differ from those of the equivalent null mutants in *P. berghei*, they are reminiscent of the phenotype of the *P. berghei* LAP4/GFP parasite described here. These discrepancies may be explained by subtle differences in the way the LAP complexes facilitate formation of the crystalloid in different *Plasmodium* spp. Accordingly, similar LAP modifications could have distinct effects on the formation of the organelle and, hence, on downstream sporogony. Our results warrant further studies of the molecular mechanisms that underlie the role of the crystalloid in sporogonic development of malaria parasites.
